# Gastric Metastasis From Lung Adenocarcinoma: An Uncommon Presentation

**DOI:** 10.7759/cureus.34587

**Published:** 2023-02-03

**Authors:** Ana Neves, Inês Mendonça, José Alberto da Cunha Marques, José Costa, Jorge S Almeida

**Affiliations:** 1 Internal Medicine, Centro Hospitalar Universitário de São João, Porto, PRT; 2 Intensive Care Medicine, Centro Hospitalar Universitário de São João, Porto, PRT; 3 Internal Medicine, Hospital Terras do Infante, Centro Hospitalar Universitário do Algarve, Lagos, PRT; 4 Medicine, Faculdade de Medicina da Universidade do Porto, Porto, PRT

**Keywords:** linitis plastica, ttf-1, lung adenocarcinoma, primary lung cancer, gastric metastasis

## Abstract

Gastric metastasis is an infrequent occurrence, especially when derived from lung adenocarcinomas. They can grossly resemble advanced gastric cancer and require comprehensive evaluations of the patients and their symptoms.

Here, we present the case of a 71-year-old patient admitted to our hospital due to intense, cramping abdominal pain. He had been previously diagnosed with a right lower lobe adenocarcinoma of the lung, which was treated in the previous year with chemotherapy and radiotherapy with good clinical response. The abdominal CT scan and the esophagogastroduodenoscopy showed a gastric infiltrating lesion resembling advanced gastric cancer. However, the biopsy showed malignant epithelial neoplasia with features of adenocarcinoma of pulmonary origin. Even though they are an uncommon finding, gastrointestinal metastases may be life-threatening and should be diagnosed as soon as possible, as the advent of molecular studies and new therapies may result in better survival rates.

## Introduction

Lung cancer is one of the most common malignancies in the world. Metastatic disease is observed in about 50% of the patients with lung cancer, with the most common sites of metastasis being the bone, liver, brain, and adrenal glands. Furthermore, peritoneal carcinomatosis from primary lung carcinoma is considered to be rare, although it can be identified in autopsy results in 2.7-16% of all lung cancer patients [1,2].

Gastrointestinal metastasis, especially gastric, from a primary lung cancer constitutes an uncommon event (0.2-0.5%) that usually occurs at advanced stages of the disease [3-5]. Although they are usually asymptomatic, the most common clinical presentation is abdominal pain [6].

Here, we present a case reported in a central hospital where a new gastric lesion was identified to be a metastatic lesion from lung adenocarcinoma.

This article was previously presented as a poster at the 19th European Congress of Internal Medicine in March 2021.

## Case presentation

A 71-year-old male patient was admitted to the hospital because of intense, cramping abdominal pain for more than one month, with marked anorexia, early satiety, and abdominal distention. He also complained about orthopnea, and worsening lower limb edema. He had no nausea or vomiting, diarrhea, palpitations, chest pain, or any other complaints. The patient reported a smoking history of 140 pack years and a drinking history of about 10 g of alcohol a day. He had no family history of cancer.

In October 2019, he was diagnosed with a right lower lobe adenocarcinoma of the lung with a Gly12Cys mutation on KRAS exon 2 (using the new-generation sequencing technique on the tumor tissue, with no additional mutations detected) and a programmed death-ligand 1 expression of 5%, stage T1cN3M0, with a 24 mm lung nodule and right broncho-hilar lymphadenopathies, with no other extrathoracic lesions (staged by positron emission tomography-computed tomography). At this time, he also had a paraneoplastic deep venous thrombosis of the right internal jugular vein and was started on proper anticoagulation. He was submitted to chemotherapy initially with carboplatin and paclitaxel (changed to pemetrexed because of limb paresthesia) and radiotherapy, both of which finished by the end of April 2020, without apparent progression. Subsequently, he started treatment with durvalumab one week before being admitted to the hospital.

According to his medical history, he also had arterial hypertension, type 2 diabetes mellitus (with atherosclerotic peripheral arterial disease, treated with metformin and sitagliptin), dyslipidemia (treated with atorvastatin and fenofibrate), obesity stage I, and obstructive sleep apnea syndrome (treated with a bilevel positive airway pressure device).

**Figure 1:**
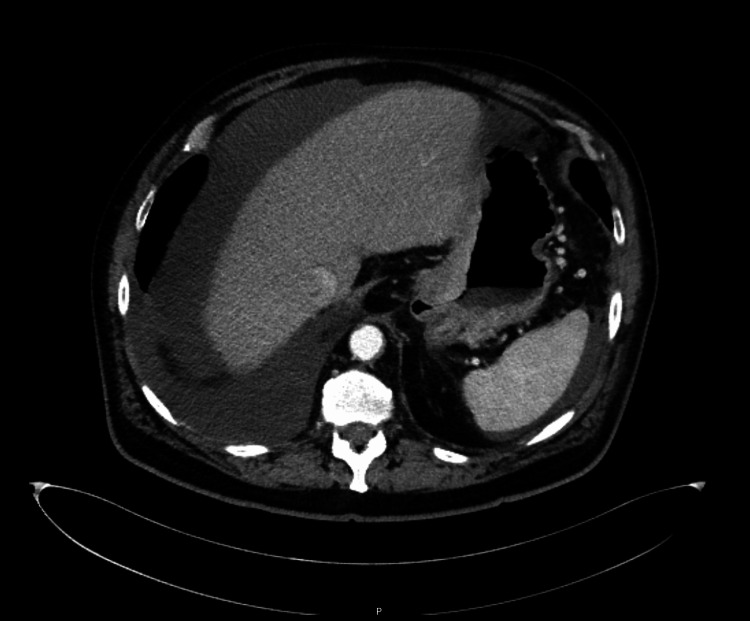
Abdominal CT scan showing marked thickening in the cardia and the region of the gastric, as well as large-volume ascites.

Because of his symptoms, he underwent a CT scan that showed a numerical and dimensional increase in mediastinal adenopathies, progression of the largest pulmonary lesion in the right lower lobe and reticulo-nodular thickening suggesting probable carcinomatous lymphangitis (Figure 1). A marked thickening was observed in the cardia and the region of the gastric antrum in relation to possible gastric neoplasia. In addition, nodular form densification of the large epiploon was noted, suggesting peritoneal carcinomatosis, with moderately large-volume ascites and pleural effusion.

After that, he underwent an esophagogastroduodenoscopy that showed a membrane of an infiltrative aspect reaching the posterior face of the body and small curvature, extending to the pyloric region, involving the pyloric region, and conditioning deformation, transposable to the endoscope. In addition, at the level of the pyloric region, he presented a clean ulcer, with a stony consistency at the edges. The lesions were biopsied, and showed malignant epithelial neoplasia with features of adenocarcinoma of probable pulmonary origin, with thyroid transcription factor-1 (TTF-1) expression.

With these results, the staging of his lung disease changed to T3cN3M1c [7]. His hospital stay was complicated by peritonitis and rapidly progressive renal failure. His therapy was optimized for major comfort, and he was transferred to a palliative care unit where he died two weeks later.

## Discussion

Because gastric metastasis grossly resembles advanced gastric cancer, it is difficult to diagnose, especially when the primary lung cancer is an adenocarcinoma [8]. Endoscopically, it usually resembles an advanced primary gastric cancer presenting with the bullseye sign or volcano-eye ulcer. An infiltrating linitis plastica pattern has been seen in only 2% of the cases of lung cancer with gastric metastasis, while it is more common in metastasis from breast carcinoma [4,8,9].

The histologic types of lung cancer that metastasize to the stomach are not well known. Some studies report squamous cell carcinoma as the most common histological presentation [2,6,10,11]. However, cases reporting the presence of lung adenocarcinoma-related gastric metastatic disease are increasing [5,9]. A final diagnosis of gastric metastasis from primary lung cancer is highly dependent on immunohistochemical results such as Napsine-A (Nap-A) and TTF-1 [5,9].

Gastrointestinal metastasis from lung cancer represents a sign of late-stage disease particularly associated with diffuse hematogenous tumor spread [4]. Tumor cells can dissect the normal gastrointestinal glands in the submucosa without evidence of preinvasive surface glandular lesions, but with the presence of diffuse lymphovascular invasion by tumor cells [3]. This leads to a late diagnosis because it can only be made after considerable growth [5].

Another study suggests that, because smokers’ gastric mucosa is more susceptible to damage, dissemination of cancer cells can occur through sputum swallowing [10].

## Conclusions

Clinicians should be aware of the possibility of gastric metastasis in patients with primary lung adenocarcinoma, and additional immunohistochemical staining for Nap-A and TTF-1 may help in distinguishing between a gastric or a lung origin. The presence of clinical gastrointestinal metastasis may be life-threatening. Comprehensive evaluations of the patients and their symptoms, with prompt investigations, are required for early detection of gastrointestinal metastasis during follow-up. With the advent of molecular studies and new therapies, better survival rates can be expected in these patients.
